# An Online Investigation of Knowledge and Preventive Practices in Regard to COVID-19 in Iran

**DOI:** 10.3928/24748307-20201130-01

**Published:** 2021-01-11

**Authors:** Mohammad Reza Heydari, Hassan Joulaei, Nooshin Zarei, Mohammad Fararouei, Zahra Gheibi

## Abstract

**Background:**

Until now, there was no available study on the knowledge and practice of the people of Iran with regard to the coronavirus disease 2019 (COVID-19) pandemic.

**Objective:**

This study aimed to investigate the knowledge and preventive practices of Iranians toward the COVID-19 pandemic.

**Methods:**

This is a cross-sectional study of 925 people who completed an online questionnaire in March 2020. The study used 21 and 14 questions, respectively, to assess the knowledge and preventive practices of the population in regard to COVID-19. Cronbach's alpha was 0.75 for the knowledge scale and 0.71 for the practice scale. To determine the importance of each independent variable in explaining the participant's practice, a multiple regression model was applied.

**Key Results:**

The results indicated a moderate level of knowledge and preventive practices in regard to COVID-19 in most of the respondents (56.8% and 56.5%, respectively). According to the multiple regression analysis, knowledge showed the highest effect on the practice of the participants (β = 0.479). The determination coefficient for the model (*R*^2^ = 0.509) also showed approximately 51% of the variance in practice was explained by gender, occupational status, knowledge, cost of hand sanitizer, and the belief in the effectiveness of using such necessities.

**Conclusions:**

Acceptable rates of knowledge and practice were observed in most Iranians. However, approximately 10% of the participants were unaware of the effective measures for preventing the infection, which can cause active transmission of the virus. In addition to considering the importance of high community awareness in prevention and isolation measures, the government should provide disinfectants and other materials at a low price to reduce the transmission, as this may lead to effective practice to break the chain of transmission of COVID-19. **[*HLRP: Health Literacy Research and Practice*. 2021;5(1):e15–e23.]**

**Plain Language Summary:**

This study sought to evaluate the knowledge and preventive practices of Iranians toward the coronavirus disease 2019 pandemic. Findings of this research demonstrate acceptable rates of knowledge and practice in most Iranians; however, about 10% of them were unaware of the true prevention practices, which can cause active transmission of the virus.

Several types of coronaviruses are known to cause respiratory infections (Z. Wu & McGoogan, 2020). Severe acute respiratory syndrome coronavirus and Middle East respiratory syndrome coronavirus (MERS-CoV) are two highly transmissible types of coronavirus that had emerged at the beginning of the 21st century (Cui et al., 2019). Coronavirus disease 2019 (COVID-19) is the most recently discovered coronavirus, and it is highly contagious in humans (Z. Wu & McGoogan, 2020). This type of virus was first diagnosed and rapidly spread from the city of Wuhan, China to the entire country of China in just 30 days (Z. Wu & McGoogan, 2020; Xiang et al., 2020).

According to the first World Health Organization (WHO) report of the novel coronavirus on December 31, 2019, of the 88,913 cases worldwide, 90% were from China (World Health Organization, 2020b). On December 16, 2020, the total number of confirmed cases of COVID-19 in the world was 71,919,725, of which 1,123,474 were reported from Iran (World Health Organization, 2020f). Since then, efforts have been made to discover a vaccine and to develop effective antiviral drugs (World Health Organization, 2020c).

The WHO declared that knowing and understanding the epidemic is the first step to defeat the virus (World Health Organization, 2020b). However, control of this highly contagious virus requires a better understanding of the behavior of the virus. Given that stopping the transmission of the virus is the most effective control strategy, improving the knowledge and preventive practices of people and communities plays a crucial role in the control of the disease (Javanian et al., 2020). From the beginning of this pandemic, free and up-to-date learning materials have been available to almost anyone interested in learning more about COVID-19 (World Health Organization, 2020c). However, although social media and the Internet have been flooded with information about COVID-19, a significant proportion of it is inaccurate and sometimes misleading, indicating a deficiency in health literacy among the general population (Spring, 2020). Hence, increasing the health literacy level of the general population will help to decrease the transmission of the virus (Paakkari & Okan, 2020).

China's successful experience in controlling the spread of the virus has also shown that one of the key factors to control the COVID-19 epidemic is to raise the health literacy of the public. For example, researchers from Zhejiang University developed and distributed easy-to-understand educational materials to inform the community about the routes of COVID-19 transmissions, its infectiousness, key measures for its prevention, and isolation rules (World Health Organization, 2020a; X. Wu et al., 2020).

Previous studies on knowledge about MERS-CoV among health care providers showed limited knowledge about the virology of MERS-CoV infection among health professionals in the southern region of Saudi Arabia (Abbag et al., 2018; Alsahafi & Cheng, 2016). A study in Anhui Province of China of 4,016 people showed high awareness of the main symptoms, transmission routes, use of masks and hand washing, and treatment of the novel coronavirus, whereas their awareness of atypical symptoms was low (Chen et al., 2020). A similar study in northern Thailand in the early period of the COVID-19 pandemic suggested poor knowledge of disease prevention and control in most of the study sample (72.9%) (Srichan et al., 2020). Additionally, 28.5% of the sample had uninformed attitudes toward disease prevention and control, and only 13.6% engaged in strong practice to prevent and control the disease (Srichan et al., 2020). The evidence showed significant effects of gender, education level, employment status, and other sociodemographic characteristics on the knowledge and practice of the participants toward coronaviruses (Aldowyan et al., 2017; Alhomoud & Alhomoud, 2017; Srichan et al., 2020; Zhong et al., 2020).

A significant improvement in a community's knowledge and practice toward COVID-19 influences the quality of surveillance, prevention, and treatment of the disease. Also, understanding the status of knowledge, attitude, and practice (KAP) in any community can help in designing more effective intervention programs to improve the health status of the community (Dauda Goni et al., 2019).

Moreover, improving social responsibility and solidarity among the population, especially among those who produce misleading and false information about COVID-19, could help to elevate health literacy (Paakkari & Okan, 2020). Because the results of previous studies indicated that a poor level of KAP causes a rapid spread of the infection in society (Asaad, 2020), and there is no available study on the knowledge and practice of the people of Iran toward the novel coronavirus, this study aimed to investigate these issues.

## Materials and Methods

### Study Design and Sample

This cross-sectional study was conducted between March 3 and 23, 2020 through an online questionnaire. According to a report from the Statistical Center of Iran, the total population of Iran at the time of the survey was about 79,926,270. Given the importance of understanding the questions, the study population included all Iranian people older than age 10 years who had access to the Internet. The age limit of 10 years was chosen because previous studies suggested children at this age can understand logical and health-related behavioral questions (Halvarsson & Sjödén, 1998; Lloyd, 1991). Therefore, with Internet penetration in Iran estimated at 69.1% in December 2017, the size of the target population of this study was 66,421,989 (World Health Organization, 2020a).

The defined minimum sample size for this KAP survey was 601 (in practice, 925 participants completed the questionnaire). The sample size was calculated using the Raosoft calculator (DaBreo & Inniss-Springer, 2016) with a confidence interval of 95%, margin of error of 4%, and a 50% response distribution for the key practice question “Are you wearing a mask?”

### Data Gathering Process

Respondents for this study were recruited through an online advertisement. For this purpose, the link to the questionnaire was shared through social platforms and apps. It was also shared with different groups such as co-workers and university students, asking them to share the link to the questionnaire with other social networks they were using. Some researchers have suggested that the distribution of the shared materials via social media is wide and fast, especially when the contents are not sensitive or private (Hu et al., 2018). Data collection was stopped after 10 days due to the completion of the required number of participants. Participation was voluntarily and the Ethics Committee of Shiraz University of Medical Sciences approved the study protocol (No.IR.SUMS. REC.1399.009).

### Study Instrument

Given the lack of a standard questionnaire to meet the objectives of this study, we designed a questionnaire based on a WHO publication on COVID-19 (World Health Organization, 2020e). The questionnaire consisted of questions regarding demographic status (including age, gender, education, and occupation of the participants). The questionnaire also included questions about the knowledge and preventive practices of the participants with regard to COVID-19.

The knowledge section comprised 6 general questions about the virus, and 17 questions on a 5-point Likert scale to measure the participant's knowledge about the prevention (*n* = 11) and transmission routes (*n* = 6) of COVID-19. To assess the practice of the participants, we used 14 questions in line with the knowledge-related items. The scores computed from these two sets of questions were equally weighted sums of the item responses. As the number of items in each scale was different, we provided two separate cut-off points (based on the quartiles of the score's distribution) for the two aspects of knowledge and practice scales. Accordingly, scores lower than 75 were regarded as inadequate, scores of 75 to 90 as adequate, and higher than 90 as good. Practice scores lower than 50 were regarded as inadequate, 50 to 60 as adequate, and more than 60 as good. In addition, due to the inadequate distribution of hand sanitizers (Humayun, 2020), gloves, and masks in Iran and many other countries, the reasons for not using such necessities were also asked to measure the causes of possible gaps between people's knowledge and practice through 9 questions using a 5-point Likert measure. These questions aimed to assess the role of lack of accessibility and high costs of masks and gloves in not practicing such important preventive measures. To facilitate the analysis and interpretation of the results, we merged and recoded those who answered *completely agree*, *agree*, and *somewhat agree* to the effect of the obstacles (i.e., unavailability and high price of the items, and belief on the effectiveness of using the materials) on their practice as “yes,” and those who answered *disagree* and *completely disagree* to the effect of the above obstacles on their practice as “no.”

To ensure the reliability of the items, the items were pre-tested on 140 people. The calculated Cronbach's alpha scores were 0.746 and 0.708 for the knowledge and practice scales, respectively (Yesilbalkan & Gencer, 2019). To examine the construct validity of the questionnaire, we used confirmatory factor analysis. The outcomes for the Kaiser-Meyer-Olkin test for the items of the knowledge and practice questions were 0.736 and 0.848, respectively. To show the suitability of the factor analysis model, Bartlett's test of sphericity generated 1,032.79 (0.000) for knowledge scale and 532.98 (0.000) for practice scale, suggesting the adequacy of the test.

### Data Analysis

The data were analyzed using the SPSS (version 21). The frequency distributions of the study variables were presented, and a comparison of the means was conducted via student *t*-test or analysis of variance to determine the differences between the groups. Linear regression analysis was also applied to determine the relationship between knowledge, cost and availability of needed materials, and practice in the bivariate analysis. To assess the adjusted associations of the above independent variable with the practice score, we ran a multiple regression model including variables that had a significant effect on the fitness of the regression model. No significant interaction was found between the key study variables (gender and age) and other independent variables included in the model. The significant level was considered *p* < 0.05 in all analyses.

## Results

A total of 925 participants completed the questionnaire. The mean (± standard deviation [*SD*]) age of the male and female participants was 40.94 years (±11.86) and 36.13 years (±9.98), respectively. Most of the respondents were employed (69.1%) and had a bachelor's degree (35.8%) (**Table 1**).

The level of knowledge about COVID-19 virus was inadequate in 9.7%, adequate in 56.8%, and good in 33.5% of the respondents (**Table 2**).

The analysis of the preventive practice scale showed that the scores were inadequate in 7.5%, adequate in 56.5%, and good in 36% of the study participants. **Table 3** shows some key results of the practice data.

The results of the independent sample *t*-test showed that the scores of the knowledge and practice of the female respondents with regard to COVID-19 were significantly better than the male respondents (*p* < .05). The dissimilarities in knowledge and practice scales according to the occupational status of the participants were also statistically significant with a higher mean in both scales (*p* < .05) for respondents who were employed. The mean of the two scales for respondents who had jobs related to health and medical sciences was also higher than in those without such occupations (*p* < .05). In addition, both the knowledge and practice scores were higher among older age groups (*p* < .05) (**Table 4**).

As **Table 4** shows, the mean of preventive practice scores was lower than the mean of knowledge scores according to all mentioned independent variables. The association between these two variables was statistically significant (β = 0.566, *p* < .05).

The high cost of hand sanitizers, gloves, and masks along with the lack of availability of these necessities were mentioned as barriers to practice (54.3% and 72.9%, respectively). The results of simple linear regression showed significant relationships between high cost (β = −0.374, *p* < .05), lack of accessibility to hand sanitizers, gloves, and masks (β = −0.355, *p* < .05), and poor practice of preventive measures.

Overall, age, gender, occupational status, education, knowledge about COVID-19, availability of hand sanitizers, gloves and masks, cost of hand sanitizers, and belief in the effectiveness of using such necessities were significantly associated with the practice of preventive measures regarding the virus (*p* < .05).

The results of the multiple regression model are presented in **Table 5**, indicating the effect of each study variable adjusted for other explanatory variables. In that regard, knowledge had the highest effect on the participant's practice of preventive measures toward the COVID-19 virus (β = 0.479).

## Discussion

The study showed adequate to good knowledge and practice toward COVID-19 among most of the participants. A similar study in China also showed good knowledge and preventive practices in regard to COVID-19 (Zhong et al., 2020).

Participants who were female, employed, or who worked in professions associated with medical and health sciences had considerably better knowledge about COVID-19. A study in Saudi Arabia also indicated a significantly greater knowledge among women and people who worked in health care professions (Asaad et al., 2020). Similarly, Zhong et al. (2020) found that being unemployed or male was related to inadequate knowledge about COVID-19.

According to the results of the current study, age, gender, occupational status, education, knowledge about COVID-19, availability and cost of hand sanitizers, gloves and masks, and belief in the effectiveness of using such necessities were significantly associated with prevention practices toward the virus. However, after adjusting for the effect of other variables, only gender, occupational status, knowledge about COVID-19, cost of hand sanitizers, gloves, and mask, and belief in the effectiveness of using such materials remained significant.

Female participants had better preventive practices toward the COVID-19 infection, a result similar to what was reported by Aldowyan et al. (2017) among girls and women in Saudi Arabia. The evidence from China also showed that the rates of wearing a mask and not going to a crowded place were higher among girls and women (Zhong et al., 2020). Another study on the same subject suggested that employment is a predictor of better knowledge (Alhomoud & Alhomoud, 2017). Also, as we observed in our study, a study in China (Zhong et al., 2020) suggested that education was not a significant predictor of preventive practices toward the COVID-19 infection.

Concern about the cost of hand sanitizers was another predictor of poor practice. Noticeably, inadequate access of Iranians to such materials was due to high cost (IRIB News Agency, 2020). This in turn could affect the practice of people and explain the gap between knowledge and practice of prevention.

In our study, participants with greater knowledge had significantly better preventive practices. It is in line with a previous study demonstrating greater use of alcohol-based hand sanitizers among participants with better knowledge (Alfahan et al., 2016). A study on MERS-COV also showed knowledge as a predictor of better practice of prevention among Muslim pilgrims (Alhomoud & Alhomoud, 2017).

## Study Limitations

As this was an online survey and the link of the questionnaire was shared through social apps and groups, approximately one-half of the respondents had a higher education level. Moreover, due to less Internet use among the older population in Iran (Statistical Center of Iran, 2017), the study had fewer participants who were older. As to be expected from a sample like this, only a few people did not believe in the effectiveness of hand washing and hand sanitizers. However, we believe the instability of the estimate did not fundamentally affect our interpretation of the results.

## Conclusion

This study demonstrated the key role of knowledge and better understanding in practicing preventive measures from the COVID-19 epidemic in Iran. Despite an acceptable level of knowledge and practice for most of Iranians, inadequate knowledge of approximately 10% of the participants suggest that the infection could continue to spread among the Iranian population and cause failure of the implemented preventive actions. Neither high cost nor low health literacy should be allowed to perpetuate this pandemic; therefore, the government, health providers, health professionals, and the media should do their part to improve the health literacy of the general population. The authorities should provide the population with clear, simple, and applicable information.

The government should also consider the affordability of the necessary materials during this pandemic. The government must provide disinfectants, hand sanitizers, masks, and gloves at a low price so that people can take action to break the chain of transmission of the infection. Establishment of an effective communication plan to inform people about healthy behaviors, prevention of infection, self-reporting, and home care of family members who are infected should also be prioritized.

## Figures and Tables

**Table 1:** Sociodemographic Characteristics of the Iranian Participants (*N* = 925)

**Variable**	***n* (%)**

Age, years	
10–19	42 (4.6)
20–29	205 (22.2)
30–49	362 (39.1)
40–50	191 (20.6)
≥51	125 (13.5)

Gender	
Female	531 (57.4)
Male	394 (42.6)

Education	
With university degree	706 (76.3)
Without university degree	219 (23.7)

Employment status	
Employed	639 (69.1)
Unemployed	286 (30.9)

Type of occupation^a^	
Not related to medical and health sciences	346 (54.1)
Related to medical and health sciences	293 (45.9)

a Only includes people who were employed when study was conducted.

**Table 2:** Knowledge Regarding COVID-19 and the Distribution of Participants' Answers (*N* = 925)

**Statment**	**Participant Answer, *M* (*SD*)**				
	**Completely Agree**	**Agree**	**Somewhat Agree**	**Disagree**	**Completely Disagree**
The incubation period of the disease can last up to several months	112 (12.1)	155 (16.8)	347 (37.5)	160 (17.3)	151 (16.3)
Older people, people with a history of certain diseases, and pregnant women are more at risk	727 (78.6)	12 (1.3)	15 (1.6)	17 (1.8)	154 (16.6)
Strong antibiotics are effective in preventing and treating the disease	24 (2.6)	411 (44.4)	271 (29.3)	174 (18.8)	45 (4.9)
People without symptoms do not need to wear a mask	212 (22.9)	117 (12.6)	192 (20.8)	79 (8.5)	325 (35.1)
Patient care does not require wearing a mask	10 (1.1)	678 (73.3)	204 (22.1)	22 (2.4)	11 (1.2)
Herbal medicine can be used to treat coronaviruses	27 (2.9)	254 (27.5)	222 (24)	303 (32.8)	119 (12.9)
Wearing a mask is recommended for everyone	111 (12)	157 (17)	365 (39.5)	98 (10.6)	194 (21)
It is necessary to wash your hands only before wearing a mask	73 (7.9)	442 (47.8)	278 (30.1)	43 (4.6)	89 (9.6)
Covering the mouth when using a mask is enough	28 (3)	447 (48.3)	348(37.6)	51 (5.5)	51 (5.5)
Avoiding contact with animals is recommended to prevent the disease transmission	26 (2.8)	38 (4.1)	100 (10.8)	270 (29.2)	491 (53.1)
Washing hands with soap and water or using hand sanitizer is effective in preventing disease transmission	14 (1.5)	5 (0.5)	6 (0.6)	142 (15.4)	758 (81.9)
A distance of 30 cm from a person who sneezes or coughs is sufficient to prevent transmission	428 (46.3)	322 (34.8)	43 (4.6)	63 (6.8)	69 (7.5)
It is not necessary to avoid touching the eyes, nose, and mouth to prevent the transmission of the disease	646 (69.8)	212 (22.9)	17 (1.8)	15 (1.6)	35 (3.8)
Not leaving the house if feeling unwell is effective in preventing the transmission of the disease	20 (2.2)	19 (2.1)	26 (2.8)	226 (24.4)	634 (68.5)
Eating raw foods is not recommended during the corona pandemic	53 (5.7)	41 (4.4)	60 (6.5)	212 (22.9)	559 (60.4)
Shaking hands and hugging have no role in transmitting the disease.	732 (79.1)	154 (16.6)	9 (1)	5 (0.5)	25 (2.7)
Coronavirus can be transmitted through contact with surfaces and objects	3 (0.3)	8 (0.9)	19 (2.1)	203 (21.9)	692 (74.8)
Coronavirus can be transmitted through sneezing and coughing	4 (0.4)	2 (0.2)	5 (0.5)	163 (17.6)	751 (81.2)
Contact with mucous membranes (nasal and oral water) can transmit the disease	0 (0)	9 (1)	17 (1.8)	155 (16.8)	744 (80.4)
The disease is not transmitted through shaking hands and hugging	669 (72.3)	184 (19.9)	9 (1)	16 (1.7)	47 (5.1)
Coronavirus can remain on the surface for several months	244 (26.4)	340 (36.8)	161 (17.4)	94 (10.2)	86 (9.3)

Note. COVID-19 = coronavirus disease 2019.

**Table 3:** Key Prevention Practices According to Gender

**Key Practice**	**Gender, *n* (%)**	
	**Male**	**Female**
Using hand sanitizers or washing hands with soap and water	385 (97.7)	518 (97.6)
Not touching eyes, nose, and mouth	338 (85.8)	478 (90.0)
Leaving the house only when necessary	370 (93.9)	513 (96.6)
Not shaking hands with, or kissing or hugging others	366 (92.9)	512 (96.4)
Wearing a mask	148 (37.6)	264 (49.7)
Disinfecting handles and surfaces	354 (89.8)	493 (92.9)
Not using a mask frequently	347 (88.1)	489 (92.1)
Avoid eating raw foods	338 (85.8)	468 (88.2)

**Table 4:** Differences in Knowledge and Preventive Practices Mean Scores According to the Studied Independent Variables

	**Knowledge**		**Practice**	

**Variable**	***M*** ± ***SD***	***p* Value**	***M*** ± ***SD***	***p* Value**

Gender		.009		.001
Female	86.79 ± 7.56		58.50 ± 4.54	
Male	85.38 ± 8.31		57.40 ± 5.35	

Education	87.33 ± 7.17	0	58.42 ± 4.66	0
With university degree				
Without university degree	82.57 ± 9.04		56.78 ± 5.54	

Age group		0		0
10–19 years	78.78 ± 9.79		53.70 ± 6.88	
20–29 years	84.57 ± 7.94		57.73 ± 5.08	
30–39 years	86.77 ± 7.57		57.89 ± 4.80	
40+ years	86.96 ± 7.63		58.49 ± 4.63	

Employment status		.001		.001
Employed	86.96 ± 7.57		58.45 ± 4.73	
Unemployed	84.49 ± 8.39		57.10 ± 5.24	

Type of occupation		0		0
Not Related to medical and health sciences	85.2 4 ±7.25		57.87 ± 4.47	
Related to medical and health sciences	89.02 ± 7.45		59.13 ± 4.94	

Cost of the items affects practice		.001		0
Yes	81.78 ± 9.63		53.95 ± 6.42	
No	86.50 ± 7.70		58.31 ± 4.69	

Using hand sanitizer is effective		.073		.017
Yes	86.33 ± 7.69		58.14 ± 4.80	
No	81.62 ± 12.81		54.48 ± 7.42	

Availability of hand sanitizers affect practice		0		0
Yes	81.31 ± 9.49		54.26 ± 5.81	
No	86.76 ± 7.52		58.48 ± 4.62	

**Table 5:** Linear Multiple Regression Data^a^ for Preventive Practices Toward COVID-19

**Variable**	**β**	***T***	***p* Value**
Age	0.038	1.364	.173
Female gender	0.086	2.003	.045
Being employed	0.173	2.492	.013
Having a university degree	−0.070	−2.363	.218
Knowledge	0.479	15.969	.001
Availability of the items affect practice	0.102	0.935	.526
Cost of the items affect practice	−0.116	−2.536	.011
Using hand sanitizers is effective	−0.325	−7.970	.001

Note. COVID-19 = coronavirus disease 2019.

a *R* = 0.509.
